# Clinical Notes

**Published:** 1892-08

**Authors:** E. B. Jackson

**Affiliations:** Houston, Texas


For Daniel's Texas Medical Journal:

### CLINICAL NOTES.

E. B. JACKSON, M. D., HOUSTON, TEXAS.

IT SHOULD be generally known that constipation is too frequently supposed to be the result of sluggish liver and atonic bowels. It is caused in not a few cases by stricture of some part of the intestinal tract following the healing of broken down gummatous or non-specific ulcers, or by adhesions established by some abdominal or pelvic inflammatory action of back date, or by malignant growth, all of which may be detected by correct history and examination. The evidence furnished by the tongue is not sufficient in many cases to give a clear understanding of this very common complaint, and the treatment begun upon the intelligence only which this organ supplies is often valueless and many times mischievous.

We are all aware that it is not safe to pronounce pregnancy until the fœtal heart is heard, or ballotment is gotten, proofs of which are rarely obtainable earlier than the fifth month. But the combined following signs are strong enough evidence to justify us, no matter what the circumstances may be, in withholding even a conciliatory hopeful opinion to the contrary: 1, Amenorrhœa; 2, Uterine globular enlargement felt per vaginam and bi manually; 3, Soft, swelled, velvety and gaping os; 4, Pigmentation of labial and vaginal mucous surfaces; 5, Puffy breasts with somewhat vascular veins, with colostrum from the nipple on pressure, and with dark projecting areola studded with papillæ; 6, The uterine souffle; 7, Morning sickness which is generally connected with cases of uterine disorder, such as versions, flexions, etc., presumably reflex as it is wont to occur upon arising, and resuming action after a night's rest.

The cause of chronic vomiting is sometimes obscure and puzzling. It may occur unconnected with any definite lesion of the stomach, liver, bowels, or uterus. Lately, two women who had gone the rounds of the London hospitals searching for cure, and who had had all sorts of things done for their wombs, came to Soho hospital, complaining of persistent vomiting, difficult and painful urination, and a movable tumor which coursed visibly up and down the side. On examination with stays off, the tumor, of kidney size and shape, was felt and could be seen moving freely when exertion to resume the upright position was made. Examination of the back revealed on the lame side loss of proper fullness and resistance just over the kidney region. Both cases being affected in the right side, as is nearly always the case in "floating kidney," the tumors were believed to be each a "detached kidney." There was no fluid of account, i. e., not enough to float the organ about, therefore, it would seem more intelligible to use the latter term in speaking of the complaint. It is worth remarking how low down in the abdomen a detached kidney may travel, and it is well to remember, when consulted on "moving, coming and going" tumors that the location and constant presence of the so-called floating kidney is practically always known to the patient, whilst "phantom tumors" of flatus come, move about, and at length disappear entirely. And the "coming and going" tumor, made by the bulging of the intestines through the recti muscles, when they are lax and separate, comes during certain positions and disappears suddenly in certain other positions, quite as mysteriously to the patient, as a flatus tumor. But the loose kidney is never lost sight of, however much it may wander about. In the two cases referred to, the treatment, outside of general measures, consisted in the application of a pad and bandage for the purpose of fixing as nearly as possible the kidney upon its normal site.

